# Case report and literature analysis of ectopic gastric glands combined with intestinal-type gastric cancer in an HP-negative background

**DOI:** 10.3389/fonc.2025.1590544

**Published:** 2025-06-05

**Authors:** Shiyu Peng, Shuxin Tian

**Affiliations:** Department of Gastroenterology, the First Affiliated Hospital of Medical College, Shihezi University, Shihezi, Xinjiang, China

**Keywords:** submucosal ectopic gastric glands, early gastric cancer, case report, a 55-year-old female, pathological examination

## Abstract

We report a rare case of intestinal-type gastric cancer combined with submucosal ectopic gastric glands in a patient without Helicobacter pylori (HP) infection. A 55-year-old female presented for a routine health check-up. Gastroscopy revealed a lesion approximately 2.0 cm in size, classified as type O-IIa+IIc, located on the posterior wall of the upper gastric body. Endoscopic biopsy indicated high-grade intraepithelial neoplasia, which promoted endoscopic submucosal dissection (ESD). Pathological examination confirmed mucosal adenocarcinoma with submucosal ectopic gastric glands. Given the association of such lesions with gastric cancer, careful diagnosis and treatment are essential. The patient remained disease-free without recurrence during a 2-year follow-up period.

Submucosal ectopic gastric glands (SHGG) refer to the abnormal proliferation of gastric glandular tissue from the lamina propria into the submucosa. SHGG is often considered a benign condition (1), typically resulting from repeated mucosal injury. However, rare cases of malignant transformation have been reported (2–23). Here, we present a case of gastric dysplasia caused by SHGG, successfully diagnosed and treated with ESD.

## Case presentation

**Figure 1:**
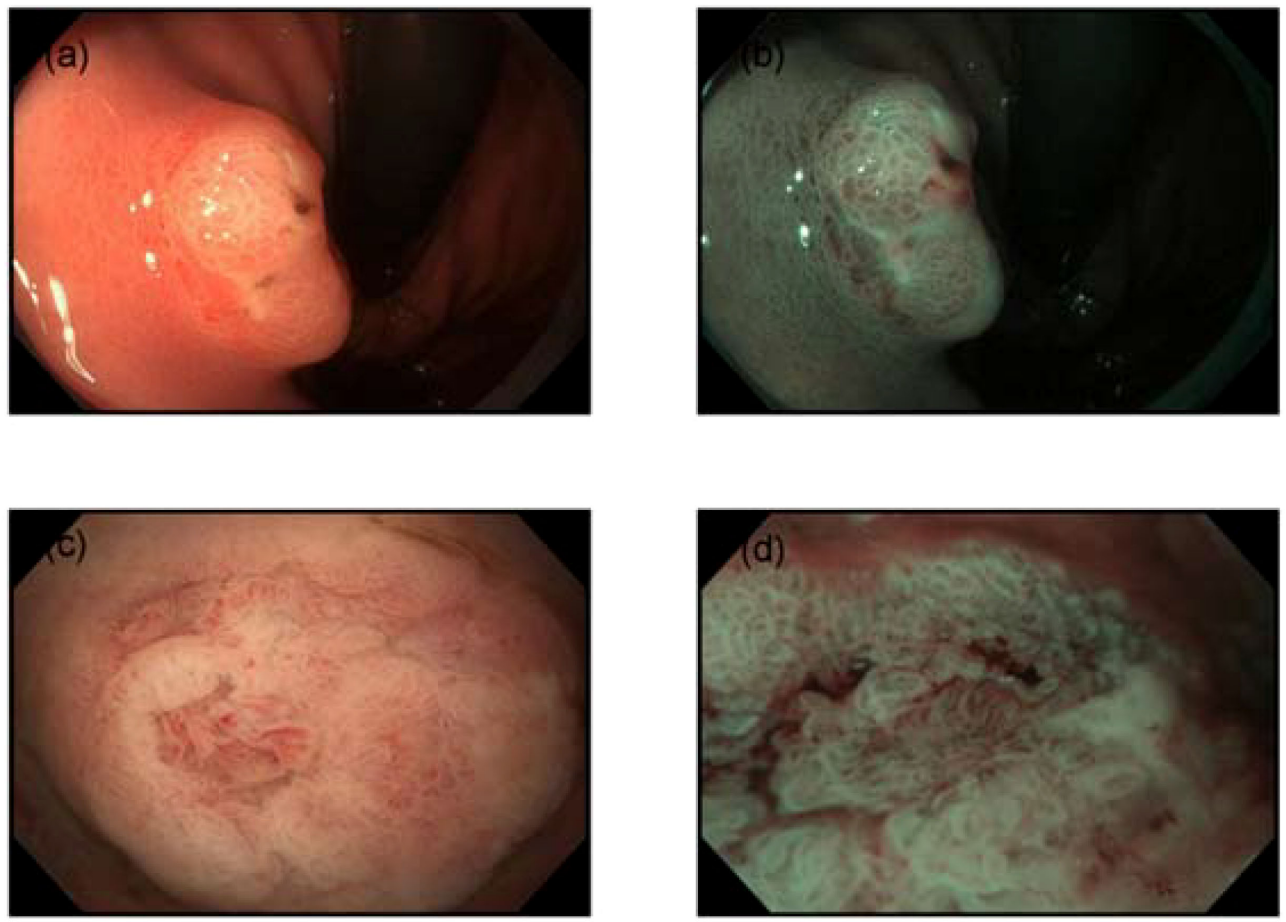
Endoscopic features. **(a)** Gastroscopy revealed a 2.0 cm subepithelial lesion on the posterior wall of the upper gastric body, with a central opening containing clear viscous fluid. **(b)** Narrow-band imaging (NBI) demonstrated villous structures within the central opening. **(c)** The mucosa surrounding and within the central pit exhibited villous structures. **(d)** Disordered and dilated microvessels were observed.

A 55-year-old female presented for a routine health check-up. Physical examination and laboratory tests were unremarkable, with no history of HP infection. No family history of malignancies or HP infection; stable family dynamics; no history of psychiatric disorders.Gastroscopy revealed atrophic gastritis and a 2.0 cm O-IIa+IIc lesion on the posterior wall of the upper gastric body. The Paris classification ‘O-IIa+IIc’ describes a raised lesion with a central depression, a pattern often seen in early-stage gastric cancers. The lesion showed a central umbilicated depression with adherent mucus, surrounded by normal-appearing mucosa (**Figure 1a**). Narrow-band imaging (NBI) showed villous structures within the central depression (**Figure 1b**). Biopsy indicated a villous-tubular adenoma with focal high-grade intraepithelial neoplasia. The gastric mucosa showed no significant atrophy, with regular arrangement of collecting venules (RAC) observed from the gastric body to the angulus. Magnified imaging revealed villous structures around and within the central pit, with focal epithelial neoplasia-like irregular structures and disordered, dilated microvessels (**Figure 1c, d**). Endoscopic ultrasound (EUS) (TGF-UC180J)confirmed intact submucosal layers, and CT scans showed no distant metastasis. The patient underwent ESD for diagnostic and therapeutic purposes.

**Figure 2:**
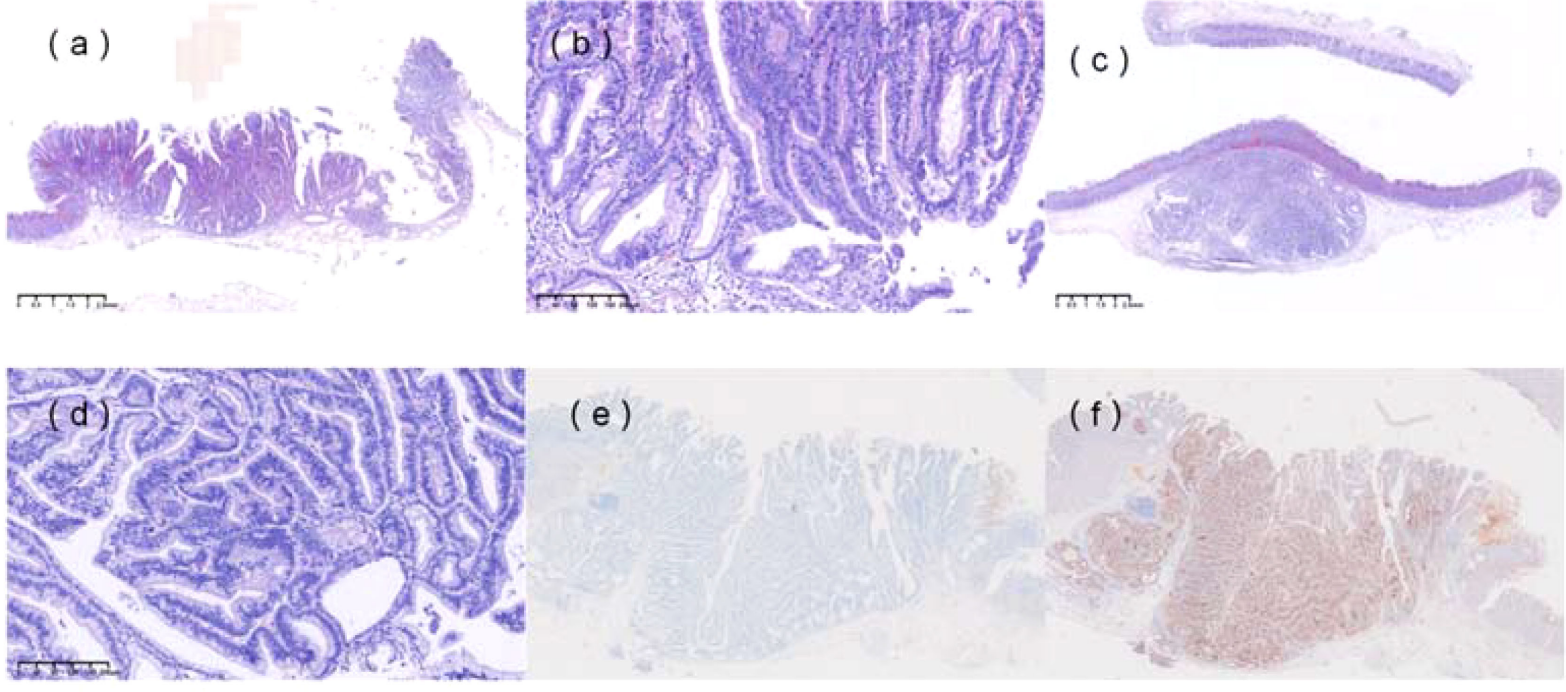
Histopathological features. **(a)** Non-neoplastic mucosa, including foveolar epithelium and pyloric glands, was observed within the submucosa, surrounded by the muscularis mucosae. **(b)** Hematoxylin and eosin (H&E) staining of the specimen (×100). **(c)** H&E staining of the resected specimen (×20) showed complete resection of the heterotopic gastric gland (HGG) component, with focal carcinomatous changes. The boundary between cancerous and non-cancerous areas was indistinct. **(d)** H&E staining of the specimen (×100). **(e)** Immunohistochemical staining revealed MUC2 positivity. **(f)** Immunohistochemical staining revealed CD10 positivity.

Pathological examination of the non-neoplastic mucosa (**Figure 2a, b**) and resected specimen (**Figure 2c, d**) revealed non-neoplastic mucosa extending into the submucosa, surrounded by the muscularis mucosae, consistent with SHGG. The boundary between cancerous and non-cancerous areas was clear. Final histopathological diagnosis confirmed well-differentiated tubular adenocarcinoma (SM1, <500 µm), with negative horizontal and vertical margins and no lymphovascular invasion. Immunohistochemistry showed MUC2+ (**Figure 2e**), CD10+ (**Figure 2f**), MUC5AC-, MUC6-, Pepsinogen-, H/K-ATPase-, CgA (focal+), P53 (wild-type), and Ki-67 (40%+). Whole-genome sequencing identified a *KRAS* mutation (exon 2, p.G12C) with a mutation frequency of 30.83%. Given the endoscopic and histological findings, the following diseases were considered and excluded, for instance, gastric inverted hyperplastic polyp (IHP), gastritis cystica profunda (GCP), primary submucosal adenocarcinoma and submucosal tumors, etc. The patient was advised to undergo a regulatory follow-up gastroscopy every 6 months for the first 2 years to monitor the potential recurrence. Given the *KRAS* mutation and background atrophic gastritis, long-term endoscopic follow-up was recommended, with further evaluation if new symptoms occurred. This study was approved by the ethics committee of The First Affiliated Hospital of Shihezi University, and written informed consent was obtained from the patient for publication and accompanying images.

## Discussion

This case represents a rare instance of intestinal-type gastric cancer in an HP-negative patient. Although HP-negative intestinal-type gastric cancers have been reported, the underlying carcinogenic mechanisms remain unclear, with potential factors including NSAID use, steroid therapy, superficial gastritis, and bile reflux. This case highlights the role of SHGG in carcinogenesis, supported by genetic analysis.

SHGG is characterized by cystic dilation of glandular structures within the submucosa, often accompanied by smooth muscle tissue continuous with the muscularis mucosae. Similar lesions include IHP and GCP, both of which have been associated with gastric cancer. A literature review identified 29 cases of SHGG combined with gastric cancer [**Table 1**: Basic Clinical Data of Patients (2–23)]. SHGG-related early gastric cancer predominantly occurs in middle-aged and elderly males, with 60% of cases located in the gastric body (**Table 2**), often infiltrating the submucosa. Immunohistochemical findings (**Table 3**) suggest that well-differentiated tumors are more common. These studies underlined the rarity of SHGG-associated gastric cancer, particularly in HP-negative patients. Consistently, the predominance of SM1 invasion and intestinal-type differentiation in line with our case, besides, the prevalence of *KRAS* mutations in SHGG lesions further supports their proliferative origin rather than inflammatory pathogenesis. There is distinct clinical and molecular heterogeneity for diagnostic and therapeutic approaches, with ESD being an effective approach for localized lesions. EUS is increasingly recognized as a valuable tool for preoperative diagnosis, with multilocular hypoechoic areas in the third layer being a characteristic feature. Diffuse SHGG with gastric cancer can complicate the assessment of tumor depth and extent. If SHGG-related cancer is confined to the mucosa or submucosa, ESD may be a viable treatment option.

**Table 1:** Basic clinical data of patients (2–23).

Case no.	Sex/Age	Symptoms	Location	Morphology	Size (mm)	Background Mucosa	Endoscopic Surface Mucosa	EUS Findings	IHP	GCP	HP	Depth of Invasion	CA199 (u/ml) / CEA (ng/m)	Surgical Procedure	Medical History
2	Male/79	Abdominal discomfort	Middle	–	25×15	Atrophy with intestinal metaplasia	Depressed lesion	–	–	–	–	SM1	–	PG	Gastric ulcer
3	Male/69	Melena	Upper	Borrmann Type I	46×30	Atrophy with intestinal metaplasia	–	–	–	+	–	SM1	Normal	TG	Gastric ulcer
3	Male/81	Vomit and bloating	Lower	Borrmann V	–	Atrophy with intestinal metaplasia	–	–	–	+	–	–	88/Normal	TG	Diabetes, hypertension
4	Male/54	–	Lower	IIa+IIc	45×35	Normal	–	Third	+	–	–	SM1	-/-	LSG	–
5	Male/45	Epigastric pain	Upper	–	5×5×4	–	–	–	–	–	+	SM1	Normal	LSG	–
5	Male/50	Epigastric pain	Lower		15×15×10	–	–	–	–	–	+	SM1	Normal	LSG	–
6	Female/50	Epigastric pain	Middle	–	35×32×18	Normal		Third	+	–	–	SM	-/-	ESD	Uterine fibroids
7	Male/70	Vomit	Lower	–	–	–	Erosion	Second	–	–	–	–	–	PG	–
8	Male/77	–	Middle	–	45×30×5		Depression	–	+	+		SM	Normal	ESD	Gastric polyp
8	Male/77	–	Middle	–	45×30×5	–	–	–	+	+	–	SM1	–	ESD	Early gastric cancer
10	Male/66	–	Middle	–	20 × 11 × 6		Normal	Third	–	–	–	SM1		LECS	–
11	Male/73	–	Middle	0-IIa+IIc	20×12×5	–	WOS, Depression	Third	–	–	–	SM1	15/3.3	PG	Diabetes, hypertension
12	Female/62	Epigastric pain	Middle	0-IIc	35×22	–	–	–	–	–	–	SM1	26.5/3.9	TG	Lumbar disc herniation
13	Male/58	Epigastric pain	Middle	0-IIa	30×20	Atrophy		Third	–	–	+	SM1	14.3/2.2	ESD+TG	Gastric cancer
14	Male/87	Decreased appetite	–	0-I+IIa	–	–	–	–	–	–	+	SM1	–	ESD	–
15	Male/71	Epigastric pain	Middle	–	–	–	–	Third	–	–	–	–	Normal/7.9	TG	Hypertension , hyperlipidemia
16	Male/70	–	Upper	–	23×15	Atrophy with intestinal metaplasia	–	–	–	–		SM1	–	ESD+TG	–
17	Male/73	–	Middle	0-IIa	17×17	Atrophy	Depression	Third	–	–	+	SM1	–	ESD	Duodenal ulcer
18	Male/85	–	–	–	28×26	–	–	–	–	–	+	SM1	Normal/4.5	PG	Colon cancer
19	Male/65	–	Middle	–	28×22	–	Depression	Third	–	–	–	SM1	6/3.8	LSG	Gastric ulcer
20	Female/80	–	Middle	–	25×20	Atrophy with intestinal metaplasia	Depression	–	–	–	+	–	Normal	PG	–
21	Female/72	–	Upper	–	20×14	Atrophy with intestinal metaplasia	–	Third	–	–	–	SM1	–	–	–
21	Male/50	–	Lower	–	15×15	Atrophy with intestinal metaplasia	–	–	–	–	–	SM1	–	–	–
21	Male/51	–	Middle		12×10	Atrophy with intestinal metaplasia	Erosion	Second	–	–	–	SM1	–	–	–
21	Male/69	–	Middle	–	10×8	Atrophy with intestinal metaplasia	Normal	–	–	+	–	SM1	–	–	–
21	Male/77	–	Middle	–	25×20	Atrophy with intestinal metaplasia	Erosion	–	–	+		SM1	–	–	–
21	Male/70	–	Middle	–	13×7	Atrophy with intestinal metaplasia	Normal	Third	–	–	–	SM1	–	–	–
22	Male/62	Acid reflux	Middle	–	–	–	Depression	–	–	–	+	–	–	ESD	–
23	Male/74	–	Middle	–	–	Atrophy	Normal	–	–	–	+	–	–	ESD	Heart disease

-, Not available or Unknown; EUS, Endoscopic ultrasound; HP, Helicobacter pylori; ESD, endoscopic submucosal dissection; TG, total gastrectomy; PG, proximal gastrectomy; LECS, laparoscopy and endoscopy cooperative surger; LSG, Laparoscopic Sleeve Gastrectomy; WOS, white opaque substance.

**Table 2:** Previous case reports of gastric carcinoma with the submucosal heterotopic gastric gland.

Total number of reported cases		N=30	%
Age	40-87 (67.1)		
Sex	Male	25	83.3
	female	5	16.7
Location	Upper	5	16.7
	Middle	18	60
	Lower	5	16.7
	Unknown	2	6.6
Size(mm)	5-46 (23.5)		
Depth of invasion	SM	2	6.7
	SM1	22	73.3
	Unknown/Other	6	20
HP	+	9	30
	–	21	70
EUS (Originating Layer of the Gastric Wall)	Second	2	6.7
	Third	10	33.3
	Unknown/Other	16	53.3
Treatment	ESD	8	26.7
	TG	4	13.3
	PG	5	16.7
	ESD+TG	2	6.7
	LECS	1	3.3
	LSG	4	13.3
	Unknown	6	20

EUS, endoscopic ultrasound; HP, helicobacter pylori; ESD, endoscopic submucosal dissection; TG, total gastrectomy; PG, proximal gastrectomy; LECS, laparoscopy and endoscopy cooperative surgery; LSG, Laparoscopic Sleeve Gastrectomy.

**Table 3:** Immunohistochemistry.

Case no.	MUC1	MUC2	MUC5AC	MUC6	CD10	Ki-67 (%)	Pepsinogen-I	H+/K+ ATPase	P53
4	–	–	+	+	–	70	–	–	+
5	–	–	+	–	–	80	–	–	+
7	–	–	+	+	–	–	–	–	–
8	–	–	–	–	+	–	–	–	–
9	–	–	–	+	–	–	+	+	–
11	–	–	+	+	–	14	+	+	+
12	–	–	–	+	–	low	+	–	–
13	–	+	+	+	–	–	+	–	–
14	–	–	+	+	–	–	–	–	–
17	–	–	–	–	–	+	–	–	+
This	–	+	–	–	+	40	–	–	+

-, Not available or Unknown.

The relationship between gastric cancer and SHGG may involve two pathways: (1) gastric cancer originating from SHGG and progressing into the mucosa, or (2) gastric cancer originating in the mucosa and extending into SHGG. In this case, the O-IIa+IIc lesion exhibited dilated vessels on the elevated surface, typically absent in submucosal invasive cancers but seen in carcinoids, fundic gland cancers, and mucosal cancers with submucosal invasion. NBI magnification revealed an irregular white opaque substance (WOS), characteristic of intestinal-type well-differentiated adenocarcinoma. The absence of stromal reaction or vascular invasion in the submucosal lesion, along with preserved mucosal architecture, suggests that the cancer may have originated in the mucosa and spread to SHGG. The presence of an activating *KRAS* mutation in SHGG supports the notion that SHGG is a proliferative lesion driven by oncogenic mutations.

*KRAS* encodes a small GTPase that acts as a molecular switch within the RAS/MAPK signaling pathway, thus involving cell growth, proliferation, and differentiation. *KRAS* is confirmed to be the most frequently mutated oncogene in various tumors, notably in colorectal cancer, pancreatic ductal adenocarcinoma, etc. Oncogenic *KRAS* mutations contribute to tumor progression not only by driving proliferation but also by modulating the tumor microenvironment. For instance, *KRAS* mutations upregulate PD-L1 expression, aiding immune escape, and activate inflammasomes like NLRP3, which further promote a pro-tumor inflammatory milieu (24). *KRAS* mutations often co-occur with other driver mutations such as TP53, PIK3CA, and APC, which can synergistically influence oncogenic signaling pathways and impact prognosis and therapeutic responses (25). *KRAS* mutations serve as key oncogenic drivers in many cancers by activating proliferative and immune-modulatory pathways, but are uncommon in gastric cancer, particularly in HP-negative cases (26). HP-negative gastric cancers follow distinct carcinogenic pathways involving genetic and epigenetic alterations such as CDH1 mutations and MSI, which contribute to their unique clinical and pathological features (27). *KRAS* p.G12C inhibitors, such as sotorasib and adagrasib, have demonstrated significant clinical efficacy, especially in *KRAS* G12C-mutant non-small cell lung cancer (28). However, monotherapy shows limited efficacy in colorectal cancer due to other resistance mechanisms (25). This suggests that combination therapies pairing *KRAS* G12C inhibitors with other approaches, such as chemoradiotherapy and immunotherapies, may help improve outcomes in gastric cancer, but high-quality clinical trials are further required.

The pathogenesis of SHGG is thought to involve chronic inflammation, such as erosion and regeneration. In this case, the background mucosa exhibited continuous atrophic gastritis extending to the antrum. Immunohistochemistry showed CD10 positivity, indicating intestinal differentiation. Previous reports have linked SHGG to HP-related chronic inflammation, with most cases involving well-differentiated adenocarcinoma. Chronic inflammation may lead to epigenetic abnormalities, such as DNA methylation, contributing to carcinogenesis. Gastric or duodenal reflux has also been implicated in SHGG development. However, this patient had no HP infection, and recent studies suggest that SHGG is not an inflammatory lesion but rather a proliferative disorder driven by frequent oncogenic mutations, particularly *KRAS*. Some other evidence suggests the possible pathogenesis in HP-negative intestinal-type gastric cancer, including genetic and epigenetic alterations, alternative environmental and host factors, epigenetic dysregulation, etc (29, 30). The absence of HP infection may delay preneoplastic lesion formation, but once genetic and epigenetic alterations accumulate can develop with aggressive features, understanding these underlying mechanisms is crucial for identifying therapeutic targets.

ESD has been successfully used for en bloc resection of SHGG-related lesions, demonstrating its feasibility as a treatment option. While SHGG may be a precursor to adenocarcinoma, the overall risk of malignant transformation is low, and resection of small SHGG lesions may not be necessary. However, larger SHGG lesions have a higher likelihood of containing dysplastic or cancerous components, warranting careful histological evaluation. Given the risk of metachronous lesions, long-term follow-up is recommended for patients with SHGG-related gastric cancer.

## Data Availability

The original contributions presented in the study are included in the article/supplementary material. Further inquiries can be directed to the corresponding author.
